# Assessment of Contributing Factors and Treatment Practices for Therapeutic Efficacy and Drug-Related Problems in Suicidal Psychotic Patients

**DOI:** 10.3390/brainsci12050543

**Published:** 2022-04-25

**Authors:** Saimon Shahzad, Sami Ullah, Zahid Nazar, Muhammad Riaz, Fazli Khuda, Atif Ali Khan Khalil, Mikhlid H. Almutairi, Amany A. Sayed, Sultan Mehtap Büyüker, Nazimuddin Khan

**Affiliations:** 1 Department of Pharmacy, University of Peshawar, Peshawar 25120, Pakistan; saimonshahzad@uop.edu.pk (S.S.); fazlikhuda@uop.edu.pk (F.K.); 2 Department of Psychiatry, Medical Teaching Institute Lady Reading Hospital, Peshawar 25120, Pakistan; drzahidnazar@yahoo.com; 3 Shafique Psychiatric Clinic, Peshawar 25120, Pakistan; riaz_shoaib@hotmail.com; 4 Department of Biological Sciences, National University of Medical Sciences, Rawalpindi 46000, Pakistan; atif.ali@numspak.edu.pk; 5 Department of Zoology, College of Science, King Saud University, P.O. Box 2455, Riyadh 11451, Saudi Arabia; malmutari@ksu.edu.sa; 6 Zoology Department, Faculty of Science, Cairo University, Giza 12613, Egypt; amanyasayed@sci.cu.edu.eg; 7 Department of Pharmacy Services, Üsküdar University, İstanbul 34664, Turkey; sultanmehtap.buyuker@uskudar.edu.tr; 8 Department of Biochemistry, Boston University School of Medicine, Boston University, Boston, MA 02118, USA; nazimkh@bu.edu

**Keywords:** suicidal behavior, suicidal ideations, contributing factors, therapeutic efficacy, drug-related problems, psychotic patients, Pakistani population

## Abstract

Suicide, a deliberate act of self-harm with the intention to die, is an emerging health concern but, unfortunately, the most under-researched subject in Pakistan, especially in Khyber Pukhtunkhwa (KPK). In this study, we aimed to identify risk factors that can be associated with suicidal behavior (SB) and to evaluate the prevailing treatment practices for therapeutic efficacy and drug-related problems (DRPs) in psychotic patients among the local population of KPK. A prospective, multicenter study was conducted for suicidal cases admitted to the study centers by randomized sampling. Socio-demographics and data on suicidal behavior were assessed using the Columbia-Suicide Severity Rating Scale (C-SSRS), socioeconomic condition by Kuppuswamy socioeconomic scale (KSES) and treatment adherence by Morisky Medication-Taking Adherence Scale (MMAS-4). Drug-related problems and the therapeutic efficacy of prevailing treatment practices were assessed at baseline and follow-up after 3 months of treatment provided. Regarding suicidality (N = 128), females reported more ideations (63.1%), while males witnessed more suicidal behavior (66.6%, *p* < 0.001). Suicide attempters were mostly married (55.6%, *p* < 0.002); highly educated (53.9%, *p* = 0.004); dissatisfied with their life and had a previous history (*p* < 0.5) of suicide attempt (SA) (20.6%), self-injurious behavior (SIB) (39.7%) and interrupted (IA) or aborted attempts (AA) (22.2%). A greater improvement was observed in patients receiving combination therapy (*p* = 0.001) than pharmacotherapy (*p* = 0.006) or psychotherapy (*p* = 0.183), alone. DRPs were also detected, including drug-selection problems (17.88%), dose-related problems (20.64%), potential drug–drug interactions (24.31%), adverse drug reactions (11.46%) and other problems like inadequate education and counseling (21.55%). Furthermore, it was also found that psychotic patients with suicidal ideations (SI) were significantly (*p* = 0.01) more adherent to the treatment as compared to those with suicidal attempts. We concluded that suicide attempters differed significantly from patients with suicidal ideations in psychotic patients and presented with peculiar characteristics regarding socio-demographic factors. A combination of therapies and adherence to the treatment provided better outcomes, and targeted interventions are warranted to address drug-related problems.

## 1. Introduction

Suicide is a growing health problem, emerging as the second leading cause of death globally, with a 75% rate in developing and underdeveloped countries [1,2]. It has the highest ratio among individuals aged 15–29 years [3]. Studies in the general population reported a prevalence of 4–12% suicide attempts, with a recurrence ratio (persons who attempt suicide again) of 50% [4]. The World Health Organization reported an increase of 60% in global suicide rates in the last few years [3].

Unfortunately, suicidal behavior remains an under-researched subject in Pakistan [4], with the only available data obtained from police and hospital records, which are not properly maintained and updated. The World Health Organization published a report on suicide in 2014, which projected that in Pakistan, there were 7085 females and 6021 males among the total 13,377 suicides, with a rate of 7.5 per 100,000 [3]. This accounts for a 2.6% increase in the rate since 2000 [3]. The WHO also estimates that for every suicide, there are at least 10–20 acts of deliberate self-harm, and attempted suicide may occur up to 20 times more frequently than completed suicide [5,6,7]. By this approximation, there may be between 130,000 to 270,000 acts of deliberate self-harm in Pakistan per annum [3].

Attempted suicide is associated with adverse, long-term outcomes, including psychiatric and medical co-morbidities, hospitalization, repeated suicide attempts, chronic stress and stigma [8,9]. Known risk factors for suicidal behaviors are largely based on studies of general populations. These include prior suicide attempts, underlying psychiatric and substance abuse, single marital status, unemployment and major life stressors [9,10,11,12,13].

Currently, one growing area of research includes the study of the correlation of psychiatric disorders and suicide attempts [14,15]. Individuals with psychiatric illnesses contribute to a significant percentage of the individuals who attempt suicide [6]. However, reliable predictors of suicidal behavior among populations with serious psychiatric disorders remain elusive. Wide-scale screening of psychotic patients has been suggested as a method of early detection of suicidal behavior [16]. Defining high-risk psychotic patients will allow clinicians to effectively screen patients for suicidal behavior and develop suicide prevention methods in clinical settings.

In continuation to that, pharmacotherapy, electroconvulsive therapy and psychotherapies are used for the treatment of suicidal patients as well as the underlying cause of disease. However, evidence still needs to be established for more effective and safer treatment options against suicidal behavior. Pharmacotherapy is often associated with drug-related problems (DRPs) such as adverse effects, interactions, poly-pharmacy, adherence problems and contraindications [17,18,19,20]. The risk of these problems increases with the number and variety of drugs. Up to 40% of hospitalized patients suffer from drug-related problems [17], which result in considerable morbidity and mortality [19]^.^ Fortunately, a substantial proportion of DRPs can be detected and prevented earlier by the efficient involvement and intervention of relevant health professionals [19,20,21,22,23,24] and by development of a standardized system for the identification, characterization and rectification of DRPs.

In light of the above facts, this study was designed (i) to assess various factors that can be associated with suicidal behavior, (ii) to evaluate the treatment efficacy and (iii) to determine the drug-related problems in the prevailing treatment practices in relieving the suicidal ideation and attempts in psychotic patients, which can further help to identify and suggest more tailored interventions.

## 2. Materials and Methods

### 2.1. Study Design

This multicenter prospective study was conducted at Lady Reading Hospital, MTI, Peshawar; Shafique’s Psychiatric Clinic, Peshawar, and Sibtain Anwar Psychiatry Hospital, Mardan. Study protocols were approved by the Ethical Approval Committee, Department of Pharmacy, University of Peshawar, through testament reference number 15/EC-18/Pharm; dated 16.10.2018. Patients were stratified on the basis of suicidal behavior, e.g., Suicidal Ideation and/or Suicidal attempt (Single and multiple suicidal attempters). Outcome variables were collected and assessed at baseline and 3 months after treatment.

### 2.2. Sample Size

The Minimum size defined for the sample was 113 psychotic patients having suicidal tendencies, determined by using the given formula (Equation (1)) [25].

Sample Size = N = Z_1−α/2_^2^ p (1 − p) /d^2^ (1)

whereas p = Prevalence (8%), Z_1-a/2_ = standard normal variate (1.96) and d = margin of error (0.05).

### 2.3. Inclusion and Exclusion Criteria

Participants were recruited at the above-mentioned healthcare centers in Khyber Pukhtunkhwa, Pakistan. The study population consisted of psychotic patients, positively diagnosed for suicidal behavior through the DSM-V criteria, aged 14–65 years, who had not taken treatment for suicidal behavior before the commencement of the study and psychotic inpatients having a lifetime history of attempted suicide, and those who provided informed consent to follow study procedures were included in the study conducted with effect from November 2018 till October 2020.

On the other hand, patients who were not ready to participate, could not answer the interview due to physical disability or cognitive impairment, were unable to communicate adequately, were taking treatment for suicidality for more than 3 days before the study and who could not provide the required data at follow up visits after 3 months were excluded from the study. 

### 2.4. Methods for Assessment

Properly trained health professionals conducted a structured face-to-face interview, consisting of internationally recognized and validated questionnaires (described below). Participants were asked about socio-demographic characteristics, including age, gender, education and socioeconomic status. 

### 2.5. Parametric Assessment

The major outcome variables were to identify risk factors that could be associated with suicidal behavior. Besides these, the improvement in suicidality and life satisfaction scores after the completion of prescribed treatment courses and treatment adherence by the patients at follow-up visits after 3 months of the treatment regimen were also assessed.

### 2.6. Suicidal Ideation and/or Attempt

The improvement or worsening level of suicidal ideation/attempt was evaluated by the mean score improvement on the Columbia-Suicide Severity Rating Scale (C-SSRS).

The C-SSRS is a semi-structured interview that measures suicide ideation and behavior [26]. The first part, the severity scale, is a 6-point ordinal scale, ranging from 1 (wish to be dead) to 5 (suicidal intent with the plan). Adolescents who denied ideation received a zero. The second part, the behavior scale, is a 5-point nominal scale that investigates interrupted, aborted and actual suicide attempts; preparatory behavior for a suicide attempt and non-suicidal self-injurious behavior. Certified trained researchers conducted the C-SSRS interviews of the psychotic subjects. An improvement was considered as a decrease in the suicidal ideation score at the time point of interest/the end-point or last measurement during treatment (in this study, after 3 months of treatment) from the baseline measurement. 

### 2.7. Contributing Factors for Suicidal Behavior

Various factors contributing towards suicidal behavior were assessed by the standardized, internationally recommended rating scales, i.e., socio-economic status through the “Kuppuswamy socio-economic scale (KSES)” [27], life satisfaction by the Satisfaction with Life Scale (SWLS) [28] and antipsychotics treatment as a potential risk by reviewing the previous medication taken.

### 2.8. Prevailing Treatment Practices

The treatment type as prescribed by their psychiatrists were categorized/ coded into 4 categories for a 3-month period of observation: (1) Psychotherapy— involved any form of cognitive behavior therapy and/or dialectical behavior therapy; (2) Pharmacotherapy—a prescription for any antipsychotic medication; (3) Electroconvulsive therapy (ECT) and (4) Combination therapy—a multiple therapy approach with any two or more therapies.

### 2.9. Drug-Related Problems

The prescriptions were evaluated for drug-related problems, including drug selection(s), dosage-related problem(s), drug-choice problem(s), potential drug–drug interaction(s), adverse reaction(s) and other associated problems.

### 2.10. Drug Adherence and Patient’s Compliance

The Morisky Medication-Taking Adherence Scale (MMAS-4) [29] was used at follow-up assessments to evaluate the level of compliance and adherence of the patients to their prescribed treatment.

### 2.11. Socio-Demographics

Baseline clinical predictors that were examined included sociodemographic characteristics, and independent variables like family history, socioeconomic condition, occupation, marital status, etc. were collected based on prior clinical and epidemiological studies of risk factors related to suicidality.

### 2.12. Analysis

Statistical package for social sciences (SPSS version 21) was used for data analysis. Descriptive statistics (frequencies, means, percentages and standard deviations) were computed. Inferential statistics, such as the chi-square test and odds ratios, were employed. For conclusive outcomes, false discovery rates were controlled/corrected using the Benjamini–Hochberg Procedure (B-H) using the formula *(i*/*m) × Q,* where: *i* = rank of *p*-value, *m* = total number of tests = 9 and *Q* = false discovery rate = 0.05 (5%).

Improvement in suicidality was assessed by comparing baseline and follow-up C-SSRS scores using a paired sample *t*-test. A significance level of 0.05 and a confidence interval of 95% were established for all analyses.

## 3. Results

### 3.1. Study Sample Characteristics

A total of 137 participants met the above-mentioned inclusion criteria for the said study. Nine subjects (7%) withdrew immediately and were not assessed. The final sample enrolled in this study comprised of 128 psychotic patients, among whom 63 (49.21%) had attempted suicide and 65 (50.78%) had active suicidal ideations without suicidal attempts. 

**Figure 1:**
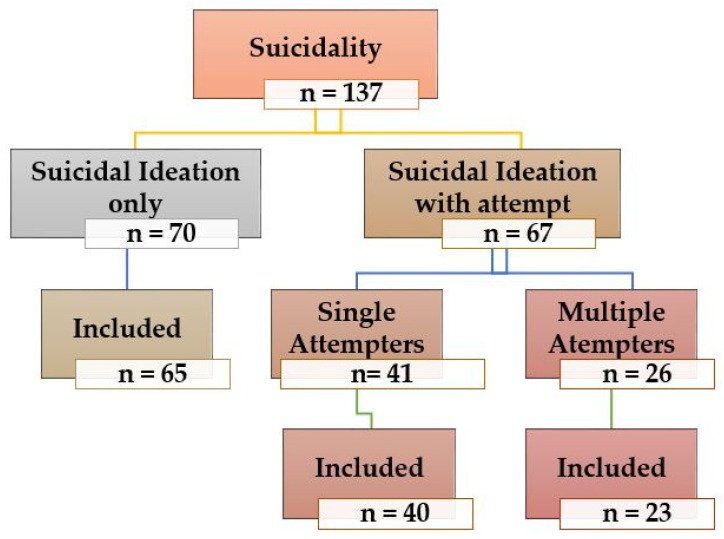
Number and sub classification categories of participants approached for recruitment and included as a study sample for assessment regarding suicidality.

Among 128 enrolled participants, 15 subjects (10.15%) failed to complete their follow-up at 3 months, and thus, 115 patients were left to complete the study from baseline to end-point. Figure 1 represents the number and sub-classification categories of participants approached for recruitment and included as a study sample for assessment regarding suicidality.

### 3.2. Socio-Demographics

The mean age of the study subjects was 23.85 years (standard deviation (SD) = 8.68 years, range of 13–65 years), and they were married (49.21%), Unemployed (28.12%) with high school or greater education (53.9%) and belonged to middle socioeconomic class (80.46%). The sample consisted of an approximately equal proportion of males (51.6%) and females (48.4%). The majority of the patients were dissatisfied (31.25%) or only slightly satisfied (22.65%) with their life and had a known history of suicidality, like previous suicide attempts (10.15%), self-injurious behavior (28.125%), interrupted/aborted attempts (10.93%) and suicidal ideations (25.78%). Besides these, a family history of suicidality was also present in a considerable proportion (35.15%) of the patients. The socio-demographic characteristics of the cases enrolled are summarized in Table 1.

### 3.3. Underlying Psychotic Diseases

**Figure 2:**
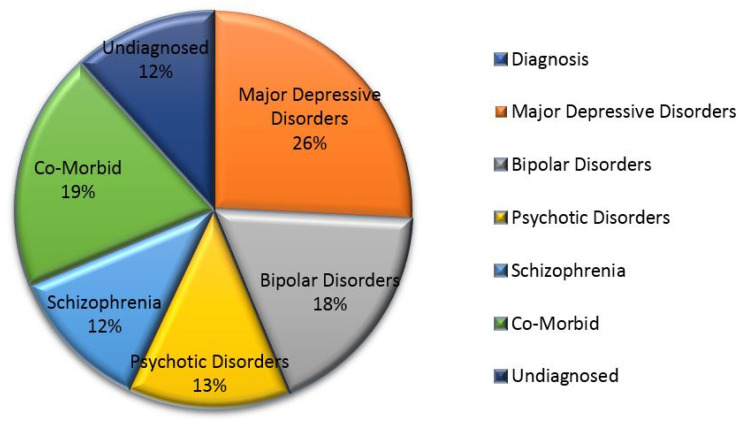
The percentage wise distribution of the underlying diagnosis of the patient with suicidality.

**Figure 3:**
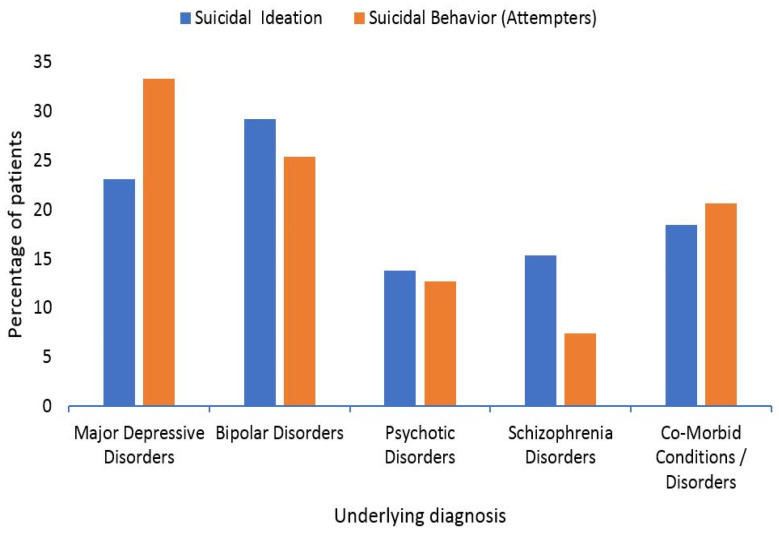
Variety and percentage wise distribution of the underlying diagnosis of the patients with suicidal behavior (Ideation and/or Attempts).

For 128 participants, psychiatric assessments/diagnoses for suicidality were performed using the DSM-V criteria by the psychiatrists in the concerned study centers. Figure 2 represents the primary psychiatric diagnosis for study participants and Figure 3 represents the percentage wise distribution of underlying diagnoses in suicidal ideation and suicidal attempt groups.

From the above figure, it is evident that in those having only suicidal ideations (*n* = 65), most prevailing percentage (23.07%) were suffering from major depression, 29.23% had bipolar disorder, 13.84% had psychotic disorders and 15.38% had schizophrenia. Conversely, in patients with suicidal attempts, the underlying diagnoses were major depression (33.33%), bipolar disorder (25.39%), psychotic disorders (12.69%) and schizophrenia (7.93%).

### 3.4. Treatment Groups 

As described earlier, therapeutic modalities were categorized into four distinct types, wherein the patient group treated with a combination therapy of electroconvulsive therapy and pharmacotherapy revealed greater improvement and showed more promising therapeutic outcomes than the psychotherapy (alone)-treated group at the endpoint assessment, i.e., after 3 months. This was evident by showing an improvement in suicidality scores on the Columbia suicide severity rating scale (C-SSRS) at follow-up visits (3 months after treatment). Data are depicted in Table 2 and Table 3.

### 3.5. Drug-Related Problems (DRPs)

Among the patients receiving medications, either in the pharmacotherapy group or combination of therapies, a total of 218 different types of drug-related problems were identified, which included drug-selection problems (17.88%), dose-related problems (20.64%), potential drug–drug interactions (24.31%), adverse drug reactions (11.46%) and other problems. The details of these problems are depicted in Table 4.

### 3.6. Treatment Adherence

Among 128 included patients, 115 subjects were able to complete the study until he follow-up assessment (after 3 months of duration). A Significant difference in treatment adherence was found in the patients with suicidal ideation versus those with suicidal behavior. Furthermore, data also depicted that, on the basis of the MMAS-4, 39 patients (33.04%) had a low adherence rate, 64 (56.52%) had an intermediate adherence rate and 12 (10.43%) had a high rate of adherence (Table 5).

## 4. Discussion

This study sought to identify characteristics and behaviors of psychotic patients associated with suicide ideations and attempts and, further, to compare the individuals who had attempted suicide and a group of psychiatric in-patients with suicidal ideations but no history of suicide attempts. These were worth-noting information to identify the potential risk factors among the vulnerable psychotic patients. The findings indicated that socio-demographic characteristics of the psychotic patients who had attempted suicide differed significantly from the subjects with suicidal ideations (Table 1). 

In this regard, a significant difference was found (*p* < 0.001, B-H = 0.01, OR: 1.892) relative to suicidality between males and female participants, indicating that the ratio of suicidal ideation was more in females (63.1%) compared to males. However, suicidal behavior, like attempts, preparatory behaviors and interrupted and aborted attempts, were witnessed the most in males (66.6%). In sum, our findings demonstrated that females and males respond differently to suicidality. 

The higher suicidal ideations in females may be explained by the fact that females are more responsible for family care, face more social pressure and exhibit poor psychological tolerance to negative life events. Women more often reported a strong negative impact on psychological well-being when they encountered adverse life events [30]. Thus, in the case of such events, they are more likely to have suicidal ideation than males but are usually reluctant to engage in a serious suicide attempt because the act is seen as violent and “masculine”. Secondly, they usually like to take others into consideration, which may become a deterrent to a suicide attempt. This can also be attributed to the ways in which females deal with problems and interact with others. They tend to share their experiences with friends, discuss their feelings, seek feedback and take advice [31]. 

Higher suicidal behaviors (attempts) in males could be explained and attributed to a few factors, including (1) that males are less likely to express and share their personal or work-related stress and problems with other people and attend psychological services compared to females [32,33,34]; (2) in Asian cultures, males are reserved in terms of the expression of sad emotions, which reflects their masculine identity, and they are expected to be strong not only physically, but also emotionally [35]; (3) males are more exposed to the use and availability of more lethal means, such as firearms and hanging methods [36,37] and to substance abuse; (4) furthermore, a male tendency to adopt avoidance strategies [38] might make it more difficult for them to cope with emotional and behavioral problems.

Therefore, there are more tendencies among males to have suicidal thinking when they are under stress. In stressful situations, they can be overwhelmed by irrational thoughts, negative emotions, feelings of hopelessness and a lack of social support and problem solving and coping skills in dealing with their stressful life events [32,33,34]. The results of this study are in line with previous studies, which showed that male youths had a considerably higher risk than females of suicide mortality and suicidality (i.e., ideation and/or attempt) [39,40,41].

Similarly, a significant difference (*p* < 0.002, B-H = 0.02, OR: 0.557) was found for suicidality regarding marital status, i.e., married versus unmarried subjects (see Table 1). There was a considerable percentage of patients (49.21%) who were married. However, previous literature reported suicide attempters as divorced/widowed [42] and less educated [41,43]. A study whose results are consistent with our findings reported that as many as 67% of the persons who committed suicide were married [44].

We also found a significant correlation (*p* = 0.004, B-H = 0.03, OR: 0.769) between educational levels and suicidality, with the least number of cases in patients educated until middle and high school and the trend gradually increasing with higher education (Table 1). The possible reasons might be that many higher education students live away from home and have less access and support from family and friends. Along with increased freedom and independence, students face greater stress from a variety of factors, such as increased academic demands, financial constraints and future insecurities. Previous studies in other populations also support our results that suicidal psychotic patients with a higher school attainment, compared with those having lower secondary school degrees, and had significantly higher ratios of suicidality [45]. This may be a considerable area to judge how those individuals with higher educational achievement may become more prone to suicide risk when facing failures, public shame and high premorbid behavior/functioning.

Our findings indicated that although the uncorrected *p*-value showed that socioeconomic status was associated with suicidal behavior, it was not significant on the B-H correction. However, socioeconomic conditions may affect the attitude towards suicidality, and the role of socioeconomic status in suicide may be worthy of further exploration. 

Life satisfaction levels also differed between the suicidal ideation and suicidal attempt groups, and it was found that those dissatisfied with their life were more prone to suicidality.

A previous suicide attempt is the strongest predictor of a future completed suicide [46]. Our study also confirmed that suicidal attempters (*p* = 0.000002, B-H = 0.005, OR: 0.390) had a history of previous suicide attempts (20.6%), self-injurious behavior (39.7%) and a history of interrupted or aborted attempts (22.2%) along with suicidal ideations (17.5%). Therefore, the past history of patients should be viewed as a marker that alerts the concerned clinicians to an increase in suicide risk in those psychotic patients [10,46].

Our study showed that those who exhibited suicidal behavior (attempts) were approximately twice as likely to report suicide attempts among family members. For some of our patients, suicides among close relatives seemed to have influenced their own actions. This condition may have been the effect of suicide contagion (imitative behavior) [47]. Similarly, it was identified that a significant positive relationship exists between the family history of suicide and the multiple attempts reported. These findings are quite similar to other reports in the literature [48].

Factors such as age and occupation did not significantly differ between the suicidal ideation and suicidal attempt groups in this study, but they have been reported as significant risk factors in studies comparing cases to community controls. These findings suggest that known risk factors of suicidal behavior may not be applicable within psychotic inpatient populations, further confirming the necessity of identifying risk factors within psychotic patients.

Unsurprisingly, the most common underlying psychiatric diagnosis was major depression disorders (26%), followed by bipolar disorders (18%), psychotic disorders (13%), schizophrenia disorders (12%) and co-morbid psychotic conditions (19%). Similar to many studies in the literature [48,49], more than 80% of suicide attempters had major depressive disorders (56–87%). Our findings reflect the consistency of the variables that were referred to in the previous literature [48,49,50]. This clearly suggests that the treatment of the above disorders should be a major component of a sound suicide prevention strategy. Suicide is a multifaceted phenomenon involving several factors, which include neurobiological, genetic and psychosocial risk factors. The complex and variable nature of suicidality requires a multidimensional prevention approach. Several studies carried out on the brain have provided important neurobiological data related to abnormalities in suicide pathogenesis. The major systems where abnormalities have been observed in suicide and nonfatal suicide attempts are the serotonergic system and the stress response systems of the noradrenergic system and the hypothalamic–pituitary–adrenal (HPA) axis [50]. 

Abnormalities in the serotonergic system have been widely implicated in suicidal behavior and ideations. This is primarily based on the studies of 5HT and its metabolite, 5- hydroxyl-indole-acetic acid (5HIAA), in the CSF [51] and blood [52] of suicidal patients; studies of 5HT receptor subtypes in the platelets of suicidal patients [53] in postmortem brains of suicide victims [54] and on serotonin neuroendocrine challenge studies [55]. The main evidence linking serotonin with suicide was derived from studies of 5HIAA, a major metabolite of 5HT in the CSF of suicidal patients. Several studies found low 5HIAA levels in the CSF of these subjects [56]. There is also an association between hypothalamic–pituitary–adrenal (HPA) axis dysfunction and suicide. The feedback regulation of the HPA axis by glucocorticoids is mediated through two different intracellular receptor subtypes, known as mineralocorticoid (MR) and glucocorticoid receptors (GR) [57]. Investigators have found an association between the dexamethasone suppression test (DST) and suicide; DST non-suppressors have been reported to be significantly more likely to commit and complete suicide than DST suppressors [58,59,60,61,62]. A significant decrease in the number of corticotrophin releasing factor (CRF) receptor binding sites in the frontal cortex of suicide victims compared with controls has also been reported [63].

Clinical application of this knowledge reflects that those serotonergic agents targeting 5HT2A receptors or 5HTT may promise a therapy for suicidal behavior. Thus, 5HT agonists may be useful in the treatment and prevention of suicidal behavior. Furthermore, targeting CRF or GR for the development of treatment for suicidal behavior may have some promise.

The severity of the suicide problem and the lack of long-term assessment of therapeutic strategies at the national and global levels demands that the efficacy of prevailing treatment regimens need to be assessed. For this, four of the considered regimen included (1) psychotherapy, (2) pharmacotherapy, (3) electroconvulsive therapy and (4) Combination therapy. The assessment was performed on the basis of improvement in mean suicidality scores on the Columbia suicide severity rating scale at follow-up visits 3 months after treatment. 

Results of the study revealed that the patients who received combination therapy (Mean C-SSRS Score Improvement = 2.14, *t*_34_ = 3.52, *p* = 0.001) and electroconvulsive therapy (Mean C-SSRS Score Improvement = 2.91, *t*_24_ = 3.98, *p* = 0.001) showed greater improvement at the end of treatment than those receiving Pharmacotherapy therapy (Mean C-SSRS Score Improvement = 1.57, *t*_32_ = 2.93, *p* = 0.006) and electroconvulsive therapy (Mean C-SSRS Score Improvement = 1.09, *t*_21_ = 1.37, *p* = 0.183). Looking through the trend, various explanations could be put forward for the improvement in suicidality. The identification of neurobiological dysfunction in the above lines suggests targets for various treatment interventions, including ECT and pharmacotherapy, that may have protective effects against suicidal behavior by correcting the dysfunctions of neurotransmitters, the neuroendocrine system and the stress response system, which have been determined for the neurobiology of suicidality [64]. Electroconvulsive therapy (ECT) is a non-pharmacologic treatment with an understudied therapeutic mechanism [14]. However, it is thought that a single ECT procedure induces a myriad of changes in the central nervous system, including a surge in circulating corticotrophins, prolactin and cortisol; changes in the immune system and increases in platelet serotonin transporters, which may be helpful to counter suicidality [65]. Pharmacotherapy in the form of antidepressants, serotonin reuptake inhibitors, antipsychotics and drugs employed to treat schizophrenia are also responsible for the correction of neurotransmitters, the neuroendocrine system and the stress response system, which, in turn, may improve suicidality, as we have discussed above that the imbalance of these system contributes to suicidality. By its nature, psychotherapy focuses on the person’s individual, subjective experience of a suicidal crisis, and there is compelling evidence that person-centered psychological treatments can significantly reduce suicidal behavior [66], particularly for patients with a history of attempted suicide. However, the lack of standard approaches and operational definitions and the paucity of standardized, reliable assessment tools to measure the suicidal outcome make it difficult to define a proper mechanism on how it can treat suicidality. Combination therapy approaches may also be effective in treating suicidality, as they can potentially add up all the benefits of various mechanisms that we had discussed until now.

Our results were consistent with previously published studies that reported the superiority of electroconvulsive therapy (ECT) [67] and indicated that ECT can be significantly more effective than pharmacotherapy [68,69,70]. In conclusion, the combination of psychotherapy, ECT and pharmacotherapy for suicidality may provide a slight advantage, whereas psychotherapy alone is not significantly effective.

The outcomes of the study have shown that the effective use of pharmacotherapy and a combination of therapies has the potential to address the increased suicidality seen in psychotic patients. However, the likelihood and higher frequency of DRPs (Table 4) in these patients need special attention and a remedial strategy.

Furthermore, it was also found that patients with suicidal ideations were significantly (*p* = 0.01) more adherent to the treatment as compared to those with suicidal attempts. Table 5 clearly indicates a positive association between treatment adherence and a decrease in suicidal behavior (attempts). A number of compliant patients who were previously categorized in the suicidal behavior group owing to an increased C-SSRS score and suicidal attempts reported a decrease in C-SSRS Scores and suicidality at their follow-up. These findings can be attributed to treatment adherence. The CSSRS Scores remained the same or even increased in those patients who discontinued or were not adherent to their treatment, which indicates that discontinuation of a prescribed treatment results in cessation of its anti-depressive, anti-schizophrenic or anti-psychotic effects and, hence, aggravates the symptoms of these disorders, among which it can enhance the risks for suicidality. Another possibility is that adherence is inversely related to levels of mental illness: as the burden of disease increases, the capability of sustaining treatment adherence decreases, which, in turn, affects the course of illness [71]. Indeed, a possibility exists that discontinuation or switching of treatments may give rise to withdrawal symptoms and rebound phenomena, whereby symptoms, including suicidality, may reoccur [72]. Nevertheless, our results are consistent with previous longitudinal studies that have identified treatment attrition as an important marker of suicide reattempts [73,74], whereas treatment compliance has been recognized as a protective factor for suicidality [75,76].

## 5. Practical Implications

The clinical implications of this study include that it can help to identify a variety of risk factors for suicidality in psychotic patients. A correct clinical/diagnostic assessment of these patients, which includes the presence of depression as a comorbid condition through an agile instrument such as the PHQ-9 [77] and determination of the presence/severity of suicidal ideation using Beck’s Scale SSI [78,79].

## 6. Strengths and Limitations of the Study

There are three key strengths of the current study. First, a rigorous qualitative methodological approach was employed, and the study comes from a country where work on the topic of suicide is scarce, a factor that contributes to a more comprehensive understanding of the problem. Second, the study involved a diverse sample of individuals with suicide of different ages; genders; occupations; educational levels and diagnoses, including in psychotic patients, and tried to highlight the particularities of this phenomenon’s manifestation in this specific population (a topic that is still quite neglected in the literature of suicidology), which contributed to the richness of the data. Third, the study results can enable professionals and researchers to understand the impact of and relationships between psychosis and suicidal thoughts and behaviors.

There were two limitations of the current study which can be taken into consideration. First, this study was limited in scope by the relatively narrow range of demographic, social and clinical correlates. Although we reported some of the most widely studied variables (e.g., age, gender, occupation and education), we could not evaluate complex variables adequately, such as the onset of illness, extent of substance abuse/dependence and adequacy of antipsychotic and antidepressant medication. Second, we relied on a clinical diagnosis at admission, and thus, replication of our observation is needed with structured diagnoses.

## 7. Conclusions

Our findings indicated that suicide attempters differed significantly from those having suicidal ideations only on the basis of factors such as gender, marital status, educational level, life satisfaction and history. The combination of therapies was advantageous, whereas psychotherapy alone was not effective (significantly). Treatment adherence translates to better outcomes. DRPs pose a serious threat to the outcomes of pharmacotherapy and combination therapy; therefore, targeted interventions are warranted.

## Figures and Tables

**Table 1:** Socio-demographic characteristics of the participants included for the assessment of suicidal behavior (N = 128); data is presented as N (%).

Category		Suicidal Ideation Only	Suicidal Behavior (Attempts)	Total	χ^2^	df	Asymp. Sig. (2-Sided) *p*-Value	B-H Value (FDR)	Odds Ratio (OR)	
		N = 65	N = 63	N = 128						
**Family History of Suicide**	Yes	14 (31.1)	31 (68.9)	45 (35.15)	10.74	1	0.001 *	0.01	3.529	
	No	51 (61.4)	32 (38.6)	83 (64.84)						
**Gender**	Males	24 (36.4)	42 (63.6)	66 (51.56)	11.33	1	0.001 *	0.01	1.892	
	Females	41 (66.1)	21 (33.9)	62 (48.43)						
**Age (years)**	13–23	20 (30.8)	17 (27)	37 (28.90)	1.64	3	0.65	0.05	1.049	1.281
	24–34	37 (56.9)	33 (58.7)	70 (54.68)					1.961	
	35–45	6 (9.2)	10 (15.8)	16 (12.5)					1.765	
	≥46	2 (3.1)	3 (4.7)	5 (3.90)					0.850	
**Socioeconomic Class**	Upper Class	10 (15.4)	3 (4.8)	13 (10.15)	5.86	2	0.05 *	0.03	3.972	1.271
	Middle Class	47 (72.3)	56 (88.9)	103 (80.4)					1.667	
	Poor	8 (12.3)	4 (6.3)	12 (9.38)					0.300	
**Occupation**	Unemployed	17 (26.2)	19 (30.2)	36 (28.12)	4.48	5	0.483	0.04	1.432	0.953
	Employed ^1^	5 (7.7)	8 (12.7)	13 (10.15)					1.789	
	Farmer	1 (1.5)	2 (3.2)	3 (2.34)					0.569	
	Student	11 (16.9)	7 (11.1)	18 (14.06)					0.503	
	House wife	16 (24.6)	9 (14.3)	25 (19.53)					1.150	
	Others ^2^	14 (21.5)	18 (28.6)	32 (25)					1.118	
**Educational Level**	Illiterate	12 (18.5)	12 (19)	24 (18.75)	17.19	5	0.004 *	0.03 ^δ^	3.600	0.769
	Primary school (1–5)	5 (7.7)	18 (28.6)	23 (17.96)					1.400	
	Middle school (6–8)	5 (7.7)	7 (11.1)	12 (9.37)					2.00	
	Secondary/High school (9–10)	4 (6.2)	8 (12.7)	12 (9.37)					0.353	
	Higher secondary school (10–12)	17 (26.2)	6 (9.5)	23 (17.96)					0.545	
	Tertiary (12+)	22 (33.8)	12 (19)	34 (26.56)					1.000	
**Life Satisfaction level**	Dissatisfied	14 (21.5)	26 (41.3)	40 (31.25)	15.75	4	0.003	0.02	0.769	0.679
	Slightly Dissatisfied	14 (21.5)	20 (31.7)	34 (26.56)					0.049	
	Neutral	11 (16.9)	1 (1.6)	12 (9.37)					0.380	
	Slightly satisfied	17 (26.1)	12 (19)	29 (22.65)					0.239	
	Satisfied	9 (13.84)	4 (6.3)	13 (10.15)					1.857	
**Marital Status**	Single	15 (23.1)	20 (31.7)	35 (27.34)	15.32	3	0.002 *	0.02	0.938	0.557
	Married	28 (43.1)	35 (55.6)	63 (49.21)					1.875	
	Engaged	2 (3.1)	5 (7.9)	8 (6.25)					0.113	
	Widowed	20 (30.8)	3 (4.8)	23 (17.96)					1.333	
**History**	No Suicidal Ideations	32 (49.2)	0 (0)	32 (25)	29.63	3	0.000002 *	0.005	0.000	0.390
	Previous Suicide Attempt	----	13 (20.6)	13 (10.15)					323	
	Self-injurious behavior	11 (16.9)	25 (39.7)	36 (28.125)					4.54	
	Interrupted/Aborted attempts	----	14 (22.2)	14 (10.93)					323	
	Suicidal ideations only	22 (33.8)	11 (17.5)	33 (25.78)					0.500	

B-H, Benjamini–Hochberg procedure; FDR, False discovery rate (5%); * *p* value < 0.05; df = degrees of freedom. ^δ^ = largest *p* value, i.e., less than its B-H critical value; ^1^. Employed includes those on job; ^2^. Others include merchants, tailors, etc.

**Table 2:** Comparative assessment of treatment efficacy for different treatment modalities through the C-SSR Scale applied at baseline and after the 3-month follow-up.

Treatment Group	Suicidality	C-SSRS		Correlation		Paired *t*-Test	
		Mean	MD	R	Sig.	*t*	Sig.
**Psychotherapy**	Baseline	5.54	1.09	0.23	0.29	1.37	0.183
	Follow-up	4.45					
**Pharmacotherapy**	Baseline	5.96	1.57	0.24	0.16	2.93	0.006
	Follow-up	4.39					
**Electroconvulsive therapy**	Baseline	5.64	2.32	0.39	0.04	3.98	0.001
	Follow-up	3.32					
**Combination therapy**	Baseline	5.8	2.14	−0.22	0.17	3.52	0.001
	Follow-up	3.65					

C-SSRS = Columbia suicide severity rating scale; MD = Mean difference; N = No. of patients.

**Table 3:** Suicidal ideation and behavior. Comparative assessment of treatment efficacy for different treatment modalities through the C-SSR Scale applied at baseline and after the 3-month follow-up in respective group patients.

Treatment Group	Suicidal Ideation						Suicidal Behavior					
	Mean C-SSRS		Correlation		Paired *t*-Test		Mean C-SSRS		Correlation		Paired *t*-Test	
	B	F	r	*p*	*t*	*p*	B	F	r	*p*	*t*	*p*
**Psychotherapy**	2.44	2.55	0.59	0.09	−0.21	0.84	7.69	5.77	0.78	0.00	6.22	0.00
**Pharmacotherapy**	3.28	3.00	0.08	0.80	0.43	0.68	7.95	5.42	−0.13	0.58	9.04	0.00
**Electroconvulsive therapy**	3.09	2.00	0.71	0.01	3.46	0.01	7.64	4.36	0.74	0.00	13.45	0.00
**Combination therapy**	4.22	2.27	−0.13	0.60	4.51	0.00	7.47	5.12	0.63	0.01	6.14	0.00

B = Baseline Score, F = Follow-up Scores, *p* = *p*-value, r = correlation, *t* = *t*-test value.

**Table 4:** Percentage wise distribution of drug-related problems (DRPs) in the treatment regimen prescribed to suicidal psychotic patients.

		N (%)	N (%)
**Drug Selection**			39 (17.88)
	Duplication	7 (3.21)	
	Wrong drug	5 (2.29)	
	Preferred/better choice of drug not given	9 (4.12)	
	Drug should have been ceased	18 (8.25)	
**Dosage-related problems**			45 (20.64)
	Inappropriate frequency	9 (4.12)	
	Inappropriate duration	11 (5.04)	
	Wrong dose/Dose changed too quickly	8 (3.66)	
	Incorrect or unclear dosing instructions	14 (6.42)	
	Dosage form inappropriateness	3 (1.37)	
**Potential Drug–drug interactions**			53 (24.31)
	Contraindications apparent	2 (0.91)	
	Minor	6 (2.75)	
	Moderate	31 (14.22)	
	Major	14 (6.42)	
**Adverse Drug Reactions**			25 (11.46)
	Symptom of an undesirable effect	19 (8.71)	
	Toxicity or allergic events	6 (2.75)	
**Other Problems**			56 (25.68)
	Incorrect Spelling of trade name	9 (4.12)	
	Inadequate education or counseling	47 (21.55)	

**Table 5:** Treatment adherence status shown by included subjects (N = 115) with suicidal ideations and behavior.

Treatment Adherence Status	Suicidal Ideation	Suicidal Behavior	Total (N)	χ^2^	df	Asymp. Sig. (2-Sided)
**High Adherence**	9	3	12	9.29	2	0.01
**Intermediate Adherence**	53	11	64			
**Low Adherence**	21	18	39			
**Total (N)**	83	32	115			
